# A Systematic Review of E-Cigarette Marketing Communication: Messages, Communication Channels, and Strategies

**DOI:** 10.3390/ijerph19159263

**Published:** 2022-07-28

**Authors:** Joanne Chen Lyu, Peiyi Huang, Nan Jiang, Pamela M. Ling

**Affiliations:** 1 Center for Tobacco Control Research and Education, University of California San Francisco, San Francisco, CA 94117, USA; pamela.ling@ucsf.edu; 2 Department of Psychology, School of Social Sciences, University of Mannheim, 68159 Mannheim, Germany; phuang@mail.uni-mannheim.de; 3 Department of Population Health, Grossman School of Medicine, New York University, New York, NY 10016, USA; nan.jiang@nyulangone.org

**Keywords:** e-cigarettes, marketing communication, marketing communication messages, communication channels, marketing communication strategies

## Abstract

Marketing plays a key role in increasing the popularity of e-cigarettes. We conducted a systematic review of the existing literature published between 2003 and 2019 in eight databases to describe e-cigarette marketing communication messages by communication channels and marketing communication strategies. Forty-one articles were included in the analysis after screening. Ten key messages were identified. Cessation and health-related benefits (each *n* = 31, 75.6%) were the most reported marketing communication messages, followed by sociability/lifestyle and use experience. The Internet (*n* = 32, 78.0%) was the most studied communication channel compared to print, TV/movie/radio, and point-of-sales (POS)/retail stores. The most studied marketing communication strategies were advertising (*n* = 28, 68.3%), followed by public relations and sales promotion. Published research studies reported consistent messages about e-cigarettes across communication channels and marketing communication strategies. Claims of smoking cessation and health-related benefits were widely identified in the existing literature. While therapeutic claims are prohibited, soft sell messages, such as social appeals, for which regulatory reach may be limited, may require educational campaigns. Internet marketing has attracted much attention, with limited studies on messages in print, TV/movie/radio, and POS/retail stores. The lack of studies of direct marketing messaging indicates a big gap between industry spending and academic research; more studies of messaging utilizing this strategy are needed.

## 1. Introduction

Electronic cigarettes (e-cigarettes) have gained increasing popularity worldwide, but there remain numerous questions related to their regulation and long-term health impact. In 2021, the global e-cigarette market reached a value of approximately 20.4 billion US dollars, and the market is projected to continue its rapid growth over the years to come, reaching 30 billion US dollars by 2027 [1]. Previous studies demonstrated that the consolidation of the e-cigarette market was accompanied by significant increases in expenditure for marketing communication [2,3,4] which is defined as “the means by which firms attempt to inform, persuade, and remind customers—directly or indirectly—about the products and brands they sell” [5]. As e-cigarette brands owned by major tobacco companies have become more prevalent, the tobacco industry’s financial resources facilitate aggressive marketing communication to consumers [6]. From 2011 to 2013, expenditures on e-cigarette marketing increased nearly 10-fold to more than $60 million in the United States [3,7]. In the first half of 2019, Juul Labs, maker of the industry-leading Juul vape brand spent $104 million on advertising [8].

The rise in popularity of e-cigarette use, positive perceptions about e-cigarettes, and interest in trying e-cigarettes are attributed to multiple factors, including aggressive marketing [9,10,11,12]. Marketing communication messages play a key role in the e-cigarette industry marketing efforts. For instance, it has been found that advertising messages emphasizing differences between e-cigarettes and cigarettes, such as “greater healthfulness”, “lower cost”, and “utility for smoking cessation” can generate more interest in e-cigarettes among smokers who had not tried e-cigarettes than messages focusing on similarities between the experience of vaping e-cigarettes and smoking cigarettes [12]. Marketing e-cigarettes as aids to achieve smoking cessation or safer alternatives to cigarettes is associated with increased e-cigarette use among young adults [10]. Marketing communication messages needs communication channels to reach target consumers. E-cigarettes are marketed through various communication channels, including the Internet, newspapers/magazines, TV/movies, and point of sales (POS)/retail stores [3,13]. It is especially notable that in recent years, social media based on the Internet technology has become an increasingly important channel to communicate e-cigarette information [14]. Collaborating with social media influencers has become a common way to influence consumer attitudes toward e-cigarettes and promote e-cigarette brands [14,15,16,17]. The e-cigarette industry also employs multiple marketing communication strategies to deliver messages, such as advertising and promotion, public relations (PR), and direct marketing [5,18,19,20,21,22]. Though much work has been performed to examine e-cigarette marketing communication messages, previous studies focused on messages conveyed in certain communication channels or certain strategies. Whether e-cigarette companies claim different messages using different communication channels and marketing communication strategies is not known.

In May 2016, the U.S. Food and Drug Administration (FDA) extended its regulatory authority to e-cigarettes [23] and gradually strengthened marketing restrictions on the industry [24]. As regulations about e-cigarette marketing have accelerated and the public interest in e-cigarettes keeps growing, a greater understanding of the e-cigarette marketing conducted by the industry is needed and fundamental to developing interventions and advising regulations to prevent youth uptake of e-cigarettes and to avoid misleading adult consumers. To fulfill these purposes, this systematic review identified major e-cigarette marketing communication messages reported in the existing literature and described the messages across communication channels and marketing communication strategies. Specifically, the current systematic review answered the following three questions based on the findings of existing research publications:

1. What are the main messages in the marketing communication conducted by the e-cigarette industry?
2. What are the main marketing communication messages delivered on different communication channels?
3. What are the main marketing communication messages delivered via different marketing communication strategies?

## 2. Materials and Methods

### 2.1. Search Strategy

**Figure 1:**
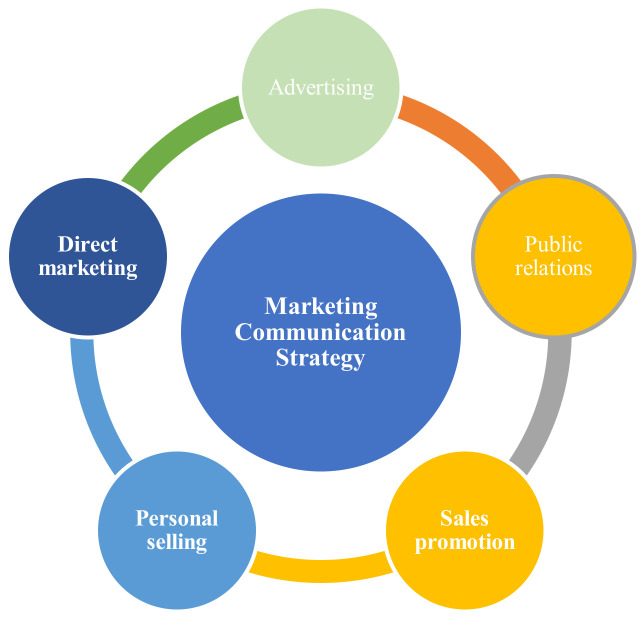
Five primary strategies in marketing communication.

Using a search strategy developed together with two librarians, we conducted a systematic search using PubMed, EMBASE, Web of Science, PsycINFO, CINAHL, Social Services Abstracts, Sociological Abstracts, and Business Source Complete databases in June 2019. Studies were limited to those published in English and in academic journals between 2003 and June 2019. There was no restriction on research methods or research design. Both quantitative and qualitative studies were included in the study. The design of the search strategy was based on a preliminary review of the relevant articles and consultation with marketing communication scholars. Each search string consisted of the following three parts: e-cigarette and its variations in terminology, communication channels, and marketing communication strategies (Figure 1), which referred to Kotler and Keller’s marketing communication mix of strategies [25] and integrated other marketing strategy literature [26,27,28].

### 2.2. Study Selection

**Figure 2:**
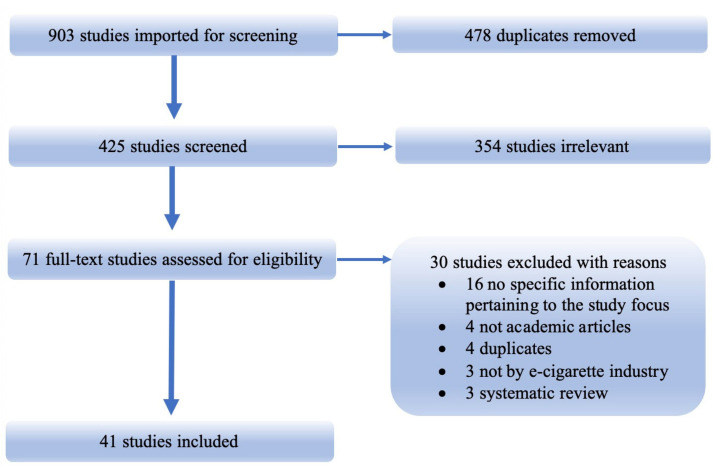
PRISMA diagram for study inclusion.

We imported all records (i.e., 903 citations) obtained through database searches into Covidence, an online software platform that streamlines the procedures of systematic reviews. In the step of importing references, Covidence can automatically identify duplicate references, and the lead authors (JL) manually checked and excluded duplicated records. Next, using predetermined criteria, two reviewers (JL and a research assistant) independently screened titles and abstracts to decide whether an article would be included for full text review. Full texts of citations judged as potentially eligible by two reviewers were retrieved. And then, the two reviewers independently screened the full texts for eligibility. Disagreements were resolved through discussions with a third reviewer (PL). Figure 2 displays the PRISMA flow diagram showing the screening process of this study. The specific selection criteria for inclusion and exclusion were elaborated on in detail as shown below:

Inclusion criteria: For inclusion in this review, research articles had to focus on e-cigarette-related marketing communication messages and at least one type of communication channel (i.e., print, TV/movie/radio, the Internet, and point of sales (POS)/retail stores) and/or at least one type of marketing communication strategy (i.e., advertising, public relations, sales promotion, personal selling, and direct marketing). Peer reviewed research articles in any scientific journal and of any study type were eligible for inclusion.

Exclusion criteria: Grey literature including dissertations, conference proceeding papers, abstracts, editorials, and commentaries was excluded. Studies that only examined the effects and expenditures of e-cigarette marketing communication or audits of e-cigarette marketing without focusing on marketing messages were excluded.

### 2.3. Data Extraction and Coding

Data extraction for the included studies was conducted by using a standardized extraction form in Microsoft Excel. For each article, the following information was extracted: author name, article title, year of publication, journal name, timeframe of studies, research method, study geographic area, marketing communication strategy, messages delivered in the marketing communication strategy, and communication channels. To ensure data extraction consistency, an author (PH) and a research assistant (RA) independently extracted data using the standardized extraction form from a test set of 16 articles (i.e., 39% of included articles), which were randomly selected. The extracted data were validated by another author (JL), and discrepancies were addressed through discussion. Both the discrepancies and consensus were documented for future reference in order to ensure the two authors extracted the remaining 25 articles in a consistent manner. Next, the extracted content was coded. The communication channel was coded into the following four categories: the Internet, print, retail stores and TV/movies [13]. Marketing communication strategy was coded into the following five categories: advertising, public relations, sales promotion, personal selling, and direct marketing. These strategies were based on Kotler and Keller’s categorization and definitions of marketing communication strategies [25] and revised after integrating further literature [26,27,28] (see Appendix A for definition of the marketing communication strategies) [5]. Development of the coding guidelines for messages was based on previous literature on e-cigarette marketing messages and the extracted content regarding messages delivered in the marketing communication strategy. We followed an iterative process of reading, testing with 10 randomly selected articles, revising, re-testing with another randomly selected 10 articles by one author (PH) and the RA until the message categories met the principle of both saturation and parsimony (Appendix B). The first author (JL) served as the third reviewer to validate the final codebook by using it to independently code a stratified random sample of 5 articles from the 41 included articles for comparison.

### 2.4. Risk of Bias Assessment

Though assessing quality and susceptibility to bias is essential in systematic reviews and researchers are faced with a large number of critical appraisal tools to choose from, there is no “gold standard” appraisal tool for any study design, nor is there consensus regarding the most appropriate items to be included in an appraisal tool [29]. No consensus exists on the ideal checklist [30]. In addition, most existing tools for quality assessment were developed for randomized controlled trials or cohort or case-control studies [31], which are not suitable for all the studies included in this review. Therefore, we adapted a checklist from the Cochrane Collaboration’s tool for assessing risk of bias [32] and the six most commonly evaluated domains in quality and bias assessment identified by Sanderson and colleagues after reviewing 86 appraisal tools [33]. Specifically, the six domains assessed in this study were (1) methods for sample selection, (2) methods for data collection, (3) methods for data analysis, (4) selective reporting, (5) conflict of interest, and (6) other sources of bias. The definition of the domains and judgment criteria are elaborated on in Appendix C.

**Figure 3:**
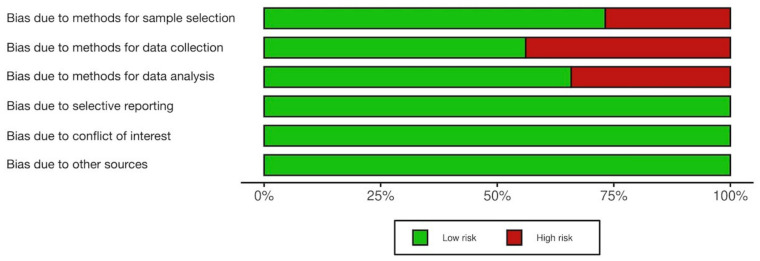
Overall assessment on the risk of bias of the included studies.

The test set of 16 articles randomly selected for data extraction training were independently assessed for risk of bias by two reviewers (PH and RA). The Cochrane Collaboration’s recommendations were adopted to judge each of the six domains as “Yes” for low risk of bias, “No” for high risk of bias, and “Unclear” for every included study [32]. Discrepancy was discussed by the two reviewers and a third reviewer until reaching consensus. The discrepancies and consensus were documented for future reference in order to make sure the two authors (PH and RA) used consistent criteria to assess the risk of bias of the other 25 articles included in the analysis. In general, the included studies were judged to have a relatively low risk of bias. No studies had a high risk of bias in terms of selective reporting, conflict of interest, and other sources of bias. Eleven studies had high risk of bias in methods for sample selection. Eighteen studies had high risk of bias in methods for data collection. Fourteen studies had high risk of bias in methods for data analysis. There were three studies with high risk of bias in two of the six assessed domains, and there were five studies with high risk of bias in three domains (Appendix D). Figure 3 shows the overall assessment on the risk of bias of the included studies.

### 2.5. Data Synthesis and Analysis

Data were summarized in a narrative synthesis and according to quantitative results. We conducted a descriptive analysis of the extracted data to describe the frequency of publication year, journal, timeframe of studies, research method, messages (including sub-messages), communication channel, and marketing communication strategy. The protocol of this review was registered at OSF Registries on 4 February 2020 (registration doi:10.17605/OSF.IO/K9BYC).

## 3. Results

The final sample consisted of 41 articles published in 21 journals from 2013 to 2019. The timeframe of the studies varied from 2008 to 2018. Sample sizes ranged from 4 spam messages/advertisements to 1.7 million tweets, with 43.9% (*n* = 18) of the sample sizes being smaller than 100, 24.4% (*n* = 10) between 100 and 1000, and 31.7% (*n* = 13) larger than 1000. Among articles that specified geographic areas, 10 articles were from the U.S., 2 studies were from Canada, and there was one study on China, Italy, and United Kingdom, respectively. The 41 articles included 34 (82.9%) content analysis studies, 4 (9.8%) secondary data analysis studies, and 3 (7.3%) observational studies. Other methods such as an interview (*n* = 2; 4.9%), network analysis (*n* = 2; 4.9%), text mining analysis (*n* = 2; 4.9%) and discourse analysis (*n* = 1; 2.4%) were also employed. 

### 3.1. Messages in Published Studies

A total of 17 sub-messages were identified and subsequently classified into 10 key messages. As shown in Table 1, cessation (*n* = 31; 75.6%) and health-related benefits (*n* = 31; 75.6%) with sub-messages of health benefits/claims, harm reduction, and healthy image were the most frequently reported messages in the studies analyzed. Sociability/lifestyle (*n* = 30; 73.2%) with sub-messages of sociability, success, and lifestyle, and use experience (*n* = 29; 70.7%) with sub-messages of enjoying vaping (everywhere) and taste/flavor also commonly used as marketing messages. Twenty-five studies (61.0%) reported marketing communication messages on price that emphasized discounts or the price advantage of e-cigarettes and 20 studies (48.8%) reported messages about product characteristics including sub-messages of product design and quality/certification. Purchase information (*n* = 13; 31.7%; e.g., information about the websites or shops to buy e-cigarettes), e-cigarette warning/disclaimers (*n* = 11; 26.8%; e.g., underage warning, product contains nicotine) and others (*n* = 5; 12.2%) were also reported (see Appendix B for details of the messages). Few studies explicitly addressed youth communication channels (*n* = 4; 9.8%; e.g., placing e-cigarettes near the popular youth items or making them visible to the youth at places such as sports venues, places with video games, school, concerts, music events, or at the movies), although other messages (e.g., low prices, attractive flavors) that also increase youth appeal were not included in this category. In addition, 20 articles (48.8%) reported messages comparing e-cigarettes with combustible cigarettes, delivering the information that e-cigarettes are more ideal in terms of being safer, healthier, cleaner, and less addictive. All the 20 papers that mentioned the comparison reported either cessation or health-related benefits. Among the 20 papers, 18 mentioned use experience, and 17 mentioned sociability/lifestyle.

### 3.2. Marketing Communication Messages across Various Communication Channels

As reflected in Table 2, the Internet was the most frequently studied communication channel (*n* = 32). In contrast, other communication channels were less studied (*n* = 4 on print, *n* = 4 on POS/retail stores, and *n* = 1 on TV/movie/radio). All the key messages were reported in the Internet studies, including cessation (*n* = 25; 78.1%), health-related benefits (*n* = 24; 75.0%), sociability/lifestyle (*n* = 23; 71.9%), use experience (*n* = 24; 75.0%), e-cigarette price (*n* = 22; 68.8%), product characteristics (*n* = 15; 46.9%), and purchase information (*n* = 10; 31.3%). Other topics (*n* = 3; 9.4%), such as information about e-cigarette marketing targeting women and e-cigarette related news and laws seldom reported by studies on other channels were also found on the Internet. Despite the limited number of studies on print media, TV/movie/radio and POS/retail stores, messages of cessation, health, sociability/lifestyle, and use experiences were identified in studies of all communication channels included in the dataset.

### 3.3. Marketing Communication Messages across Various Marketing Communication Strategies

As shown in Table 3, advertising, public relations, and sales promotion were mostly studied with *n* = 28, 10, and 10, respectively. Only one paper studied e-cigarette marketing communication messages through the strategy of personal selling. No paper reported messages via direct marketing. For strategies of advertising and public relations, the most frequently reported messages in published studies included cessation, health-related benefits, sociability/lifestyle, and use experience. Moreover, 90.0% of public relations studies (*n* = 9) reported the message of sociability/lifestyle, making it the most commonly reported type of messages in public relations studies. Sales promotion studies reported some messages with a different frequency. Though messages of cessation, health-related benefits, sociability/lifestyle, use experience, product characteristics, youth, and warnings/disclaimers were reported, price was the most reported message in sales promotion (*n* = 9; 90.0%), followed by purchase information (*n* = 3; 30.0%). While advertising studies, public relations studies, and sales promotion studies reported almost all the key messages, there were only three messages reported in the personal selling study. They were cessation, health-related benefits, and sociability/lifestyle.

## 4. Discussion

Despite clinical trials finding that e-cigarette use can assist with smoking cessation, population studies of e-cigarettes for smoking cessation have not found benefits [34,35,36]. The FDA has not approved e-cigarettes as a smoking cessation aid [37]. However, this review found that smoking cessation was the most reported marketing communication message in the existing literature. Similarly, though some studies reported decreases in symptoms or biomarkers of harm with e-cigarette use compared to smoking cigarettes, the data on long-term health consequences are inconclusive [38,39,40]. Even so, health claims were reported in more than three quarters of the included studies. It should be noted that when these studies were conducted, there was less evidence on either the effectiveness of e-cigarettes for smoking cessation or harm reduction compared to cigarettes than now. Studies of e-cigarette marketing messaging suggest that claims of e-cigarette benefits exceed the scientific evidence; more surveillance and strengthening of the regulation of health claims are needed. The marketing studies also reported messages about sociability/lifestyle, use experience, pricing, and product characteristics, which were consistent with the main themes identified in e-cigarette-related media coverage [41,42]. This may indicate that media coverage and e-cigarette marketing communication reinforce each other. It is particularly worth noting that while regulations can limit certain marketing messages such as cessation efficacy and health claims, it is difficult to regulate soft sell messages such as social acceptance, sexual appeal, successful self-image, and enjoyable use experience. The frequency of these messages reported in the literature suggests the need for educational campaigns to counter the effect of heavily disseminated soft sell marketing messages.

This study also found that among the four communication channels, i.e., print, TV/movie/radio, the Internet, and POS/retail stores, the Internet was the most studied channel (78.0% of included papers). While this finding corresponds with the growing presence of e-cigarette marketing on social media platforms [43,44,45], it indicates a large gap between the amount of marketing expenditure on print and TV and a dearth of study in terms of these channels. Data from Kantar Media, which provide information on US advertising expenditure, showed that in terms of the e-cigarette promotional spending in 2008–2013, print was the dominant channel, followed by TV [4]. Though a decline in traditional advertising venues occurred during 2014–2017, which may reflect a shift towards social media, the level of expenditure for print advertising was highest irrespective of year [20]. However, the tremendous marketing spending on traditional media did not receive commensurate academic attention. This may be explained by the easier access to the Internet data compared to other communication channels, but it also highlighted the need to delve into the marketing on traditional media to gain a more comprehensive understanding of marketing communication messages. Despite the limited number of studies on non-Internet marketing communication channels, this review observed that cessation, health-related benefits, sociability/lifestyle, and use experience were the most reported messages across all studies of communication channels, consistent with a scoping review of messages in e-cigarette promotion and discussions on social media [46]. This consistency may imply that the industry’s marketing communication messages greatly influenced and aligned with the e-cigarette discussion and promotion on social media. However, a considerable portion of our included studies were about social media, although the scoping review included social media content from a wide variety of information sources, including media, government, not for profit organizations, and public health communities, while our review focused on marketing communication messages from the e-cigarette industry only.

Cessation, health-related claims, sociability/lifestyle, and use experience are generally the most frequently reported messages in almost every study of communication channels and marketing communication strategies. However, the small number of studies on marketing communication messages delivered through POS/retail stores (*n* = 4) is notable. To obtain a more comprehensive understanding about e-cigarette marketing, more studies of marketing in the POS/retail environment are needed. In terms of marketing communication strategies, the most noticeable exception to this pattern was that the predominant message in studies of sales promotion was related to price advantage/discount. This may be because sales promotion directly correlates with the primary objective of creating an immediate sale [47]. Additionally, we found that among the five strategies, advertising, sales promotion, and public relations received most academic attention, while limited studies addressed personal selling, and we found no studies on direct marketing. Direct marketing has been an important channel for tobacco companies [48], and 16 million (7.1%) US adults reported receiving e-cigarette promotion via mails or emails in 2013–2014 [21]. Exposure to e-cigarette direct marketing promotion is associated with e-cigarette use [21]. Therefore, studies of marketing communication messages delivered in direct marketing promotion are warranted. Another less studied strategy is personal selling. Though there has been one study on personal selling, the only three messages identified in this study were cessation, health-related claims, and sociability/lifestyle. So, overall, this study identified a consistency of messaging across communication channels and marketing communication strategies to convey unified information [49]. When consistent e-cigarette marketing communication messages are disseminated on various communication channels and via various marketing communication strategies, they reinforce each other and gradually shape perceptions and attitudes toward e-cigarettes. In contrast, public health messaging about e-cigarettes has been conflicting and controversial [38,39,40,50,51], which may impair efforts to address e-cigarette marketing messages.

Most of the research studies included in this review were from the United States, and studies from other places were few. Though this may be related to the study design that only English publications were included, English speaking countries other than the US were still much less studied. Another possible explanation is that North America is by far the largest e-cigarette market in the world [52], so it attracted most academic attention. However, the e-cigarette market has been flourishing globally, so additional research on e-cigarette marketing communication in different countries is needed. It would be particularly interesting to compare e-cigarette-related marketing communication messages among countries with different e-cigarette marketing policies, providing natural experimental data that might shed light on effective e-cigarette marketing regulations. In terms of research methods, the majority of the published studies applied quantitative research methods. More qualitative research is called for, including document analysis, which is a powerful method by which to reveal the industry perspective and marketing objectives to complement content analyses [53].

This study has a few limitations. First, this study was based on research publications on e-cigarette marketing communication. Although these studies provided insight into marketing communication by the e-cigarette industry, we cannot assume that these studies reflected the full picture of the industry’s practice of marketing communication. Second, the review only included papers in English. Therefore, this review cannot reflect the message-focused marketing communication research on a global scale. Third, findings about e-cigarette marketing communication messages in different communication channels and marketing communication strategies are limited due to the small number of articles on certain communication channels and strategies.

## 5. Conclusions

This study provided a systematic review of studies of e-cigarette marketing communication messages, which contributes to an integrative perspective on various communication channels and different marketing strategies to the extant literature. We found many studies reported finding messages regarding smoking cessation, health-related benefits, sociability/lifestyle, and user experience emphasizing the ability to use the product anywhere and taste/flavor. Since e-cigarettes are not approved smoking cessation therapeutic devices, cessation efficacy and health claims should be subject to increased monitoring and regulation. Our synthesis of the studies also found that messages were highly consistent across different communication channels and marketing communication strategies. These findings suggest that public health communities may need to strengthen the integration of educational messaging to balance the relatively unified positive e-cigarette messaging from the e-cigarette industry. In addition, we found the Internet was the most studied communication channel, which aligns with the increasing social media marketing by the e-cigarette industry, but we observed that despite large expenditures on traditional media, little commensurate research on these channels has been conducted, suggesting a gap in the research literature. Specifically, we found a lack of research on direct marketing messaging in contrast to the amount of exposure reported by consumers to this marketing communication strategy. Therefore, more studies are warranted on messages in print, TV/movie/radio, POS/retail stores, and on direct marketing in order to facilitate a more comprehensive understanding of e-cigarette marketing communication.

## Figures and Tables

**Table 1:** Frequency of marketing communication messages reported in published studies (*n* = 41).

Key Message	Sub-Message	*n* (%)	
Cessation	Smoking cessation	31 (75.6)	31 (75.6)
Health-related benefits	Health benefits/claims	25 (61.0)	31 (75.6)
	Harm reduction	23 (56.1)	
	Healthy image	2 (4.9)	
Sociability/lifestyle	Sociability	26 (63.4)	30 (73.2)
	Success	9 (22.0)	
	Lifestyle	18 (43.9)	
Use experience	Enjoying vaping (everywhere)	21 (51.2)	29 (70.7)
	Taste/flavor	18 (43.9)	
Product characteristics	Product design	12 (29.3)	20 (48.8)
	Quality/certification	13 (31.7)	
Price	Discount or price advantage	25 (61.0)	25 (61.0)
Youth	Youth-resonant information	4 (9.8)	4 (9.8)
Warnings/disclaimers	Health disclaimers/warnings	6 (14.6)	11 (26.8)
	Age restriction	9 (22.0)	
Purchase information	Information for easy purchase	13 (31.7)	13 (31.7)
Others	Miscellaneous information	5 (12.2)	5 (12.2)

**Table 2:** Frequency of marketing communication messages across communication channels in published studies (*n* = 40 *).

Key Message	Print (*n* = 4)	TV/Movie/Radio (*n* = 1)	Internet (*n* = 32)	POS/Retail Stores (*n* = 4)
Cessation	2 (50.0%)	1 (100.0%)	25 (78.1%)	2 (50.0%)
Health-related benefits	3 (75.0%)	1 (100.0%)	24 (75.0%)	2 (50.0%)
Sociability/lifestyle	3 (75.0%)	1 (100.0%)	23 (71.9%)	2 (50.0%)
Use experience	3 (75.0%)	1 (100.0%)	24 (75.0%)	1 (25.0%)
Product characteristics	1 (25.0%)	1 (100.0%)	15 (46.9%)	2 (50.0%)
Price	1 (25.0%)	1 (100.0%)	22 (68.8%)	0 (0.0%)
Youth	0 (0.0%)	1 (100.0%)	1 (3.1%)	2 (50.0%)
Warnings/disclaimers	1 (25.0%)	1 (100.0%)	7 (21.9%)	1 (25.0%)
Purchase information	1 (25.0%)	1 (100.0%)	10 (31.3%)	0 (0.0%)
Others	1 (25.0%)	0 (0.0%)	3 (9.4%)	0 (0.0%)

* One of the 41 articles did not specify the communication channels.

**Table 3:** Frequency of marketing communication messages across marketing communication strategies in published studies (*n* = 36 *).

Key Message	Advertising (*n* = 28)	Public Relations (*n* = 10)	Sales Promotion (*n* = 10)	Personal Selling (*n* = 1)
Cessation	22 (78.6%)	4 (40.0%)	2 (20.0%)	1 (100.0%)
Health-related benefits	22 (78.6%)	4 (40.0%)	2 (20.0%)	1 (100.0%)
Sociability/lifestyle	21 (75.0%)	9 (90.0%)	1 (10.0%)	1 (100.0%)
Use experience	21 (75.0%)	6 (60.0%)	2 (20.0%)	0 (0.0%)
Product characteristics	16 (57.1%)	3 (30.0%)	2 (20.0%)	0 (0.0%)
Price	14 (50.0%)	4 (40.0%)	9 (90.0%)	0 (0.0%)
Youth	4 (14.3%)	0 (0.0%)	1 (10.0%)	0 (0.0%)
Warnings/disclaimers	7 (25.0%)	2 (20.0%)	1 (10.0%)	0 (0.0%)
Purchase information	9 (32.1%)	1 (10.0%)	3 (30.0%)	0 (0.0%)
Others	4 (14.3%)	1 (10.0%)	0 (0.0%)	0 (0.0%)

* Five of the 41 included articles did not specify marketing communication strategies.

**Figure A1:**
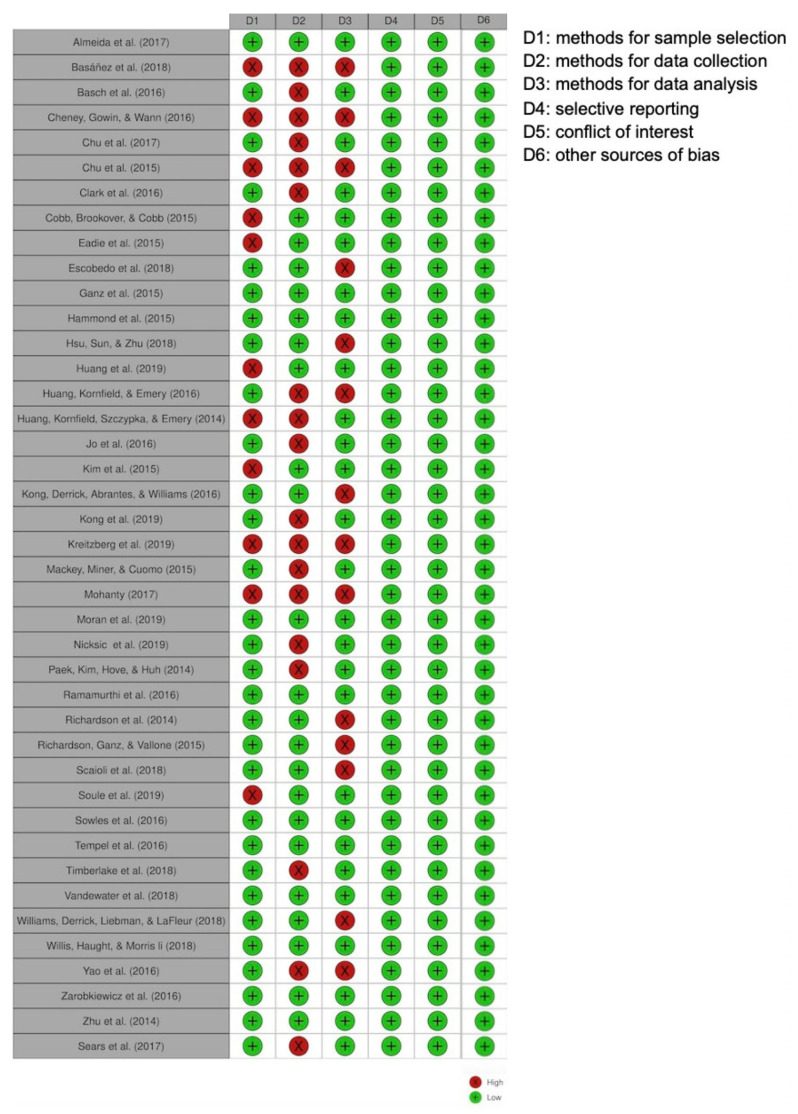
Risk of bias assessment of included studies (*n* = 41).

## Data Availability

Not applicable.

## Appendix A

**Table A1:** Marketing communication strategy definitions and examples.

Strategy	Definition	Example
Advertising	Paid and non-personal presentation and promotion of ideas, goods, or services by an identified organization	Ads in print media, radio, billboards, television, online social media, mobile, etc.
Public relations	Two-way strategic communication to promote, maintain and protect the goodwill and image of the company and its product or service in the market and present them in a positive light	Publicity such as press releases and news, sponsorship, creating positive Word-of-Mouth (WOM), etc.
Sales promotion	Short-term incentives to encourage customers to buy a product/service	Coupons, discount, premiums, samples, price packs, etc.
Personal selling	Face to face interactive presentation by the sales force to engage the customers, sell the product/service and build good customer relationships	Sales recommend products to customers in stores
Direct marketing	Directly engage with targeted individuals or groups by sending an offer, announcement, reminder, or other items to obtain an immediate response and build long-lasting customer relationships. It includes the concepts of direct-mail marketing and direct and digital marketing	Organizations directly communicate with end users through post E-mails, telephone, fax, text messages, catalogue, brochure, and promotional letter. In internet or social media: can be a link to shop/company website (but not related to sales promotion or public relations activities)

## Appendix B

**Table A2:** Marketing communication messages, sub-messages, and examples.

Key Message	Sub-Message	Examples
**Cessation**	**Smoking cessation**	E-cigarette is promoted as effective cessation device and can help quit or reduce cigarette/tobacco smoking (e.g., “switching”, “alternative to smoking/quitting”, “replacing”, “remedy to nicotine addiction”). 1. A direct claim of e-cigarettes as an effective quitting aid; 2. An indirect claim (e.g., a featured customer testimonial) of e-cigarettes as an effective quitting aid.
		Reduce risk to your health/quit smoking; reduce withdrawal symptoms
**Health-related benefits**	**Health benefits/claims**	E-cigarette is a medical product
		E-cigarette alleviates specific medical condition
		E-cigarette reduces stress
		E-cigarette smoking gains no weight
		Short-term health (e.g., no smoker’s cough, ability to breathe better)
		Long-term health (e.g., live longer, no risk of cancer)
		E-cigarette is not a cigarette (or not a tobacco product)
		E-cigarette is healthy/safe/harmless
		E-cigarette is not addictive, or it is perceived that nicotine/ingredient in e-cigarettes is not additive
		Physician/expert endorsement: Indicates that the product/ad is endorsed by a physician (e.g., “doctor recommended”, physician narrator, recommended by WHO as NRT (false claim))
	**Harm reduction**	For self: presents a message that e-cigarette is less harmful or safer for vapers themselves (e.g., light/low tar, no tar, free of tar and other carcinogenic substances, presence of less toxins than cigarettes, e-cigarette reduces risk of tobacco-related diseases)
		For others: presents a message about reduced risks or reduced harm or safer for others (e.g., product does not expose others to secondhand smoke, no secondhand smoking concerns based on the reduced harm)
		Harm reduction due to no environmental smoke: suggests that the product is greener or more environmentally friendly than cigarettes (e.g., “no secondhand smoke”, “no ash”, “no smoke”)
	**Healthy image**	Image portrayal without text claim
**Sociability/Lifestyle**	**Sociability**	Social acceptance: associates the product with increased social acceptability, higher standing in society, and celebrating/praising others using the product (e.g., socially accepted, products do not bother non-smokers based on the social concern)
		Sex/romantic: depicts greater ability to engage in romantic/sexual encounters, increased sex appeal/ability to attract desired sex, contains sexual innuendo
		Sharing: 1. Experience: Sharing personal experiences regarding e-cigarette. 2. Feelings: Sharing video creator’s feelings after vaping e-cigarette
		Individuality/freedom/independent: associates product use with the consumer being their “own person”, taking control of their life or aspects of their life, having no restrictions on their life or their activities (e.g., having a free spirit, increase personal freedom, increase control of experience)
		Fun: fun with friends, hip, enjoyment, happiness
		Cool: being “cool”, contests, adventures
	**Success**	Self-image boosting as responsible, successful in appearance or actions Increase social status Enhance self-esteem and self-confidence
	**Lifestyle**	Party: nightlife, celebrities, etc.
		Music: Uses background music, contains background special effects or sounds effects (e.g., a car horn, door slamming, clapping)
		Sports: References sports or games (e.g., a baseball game, basketball game, video games)
		Holiday: references a holiday (e.g., Christmas, Valentine’s Day, New Year’s), relaxing
		Humor: Uses humor/humorous noncritical joking to sell the product (e.g., lighthearted, inclusion of joke, sarcasm)
		Modern & trendy: fashionable, revolutionary, realistic smoking experience
		Vaping as habit instead of addiction
**Use experience**	**Enjoying vaping (everywhere)**	Vaping freely: the product can be used to circumvent smoking restrictions (e.g., “smoke free laws”, “smoke-free rules”, “clean indoor air regulations” or “smoking bans”) or emphasizes the ability to use the product anywhere (e.g., offices, planes, restaurants, bars)
		Use and enjoy: How to use/modify/enjoy e-cigarette
	**Taste/Flavor**	Taste references the taste of the product (e.g., highlighting satisfaction, pleasure, freshness, long-lasting taste, aroma) 1. Taste (general): Taste information of e-cigarette in general 2. Taste (specific brand): Taste information of e-cigarette specific brand
		Flavor Indicates whether a flavored product is being advertised
**Product characteristics**	**Product design**	Technology/device: Highlights the technology and/or device characteristics (e.g., battery type/size, revolutionary, break-through, sophisticated, electronic capability, “modern or revolutionary way to smoke”, “the product is rechargeable”)
		Product information: about e-cigarette product contents (including ingredients)
		Design information: about e-cigarette appearance (e.g., modern and technologically advanced, “the product is new”)
		Uniqueness: the product is unique
	**Quality/Certification**	Quality: company ensures the excellence and quality of the electronic device and the liquid solutions, choosing valuable raw materials and making steady and accurate controls (e.g., highlighting organic or “pure” qualities of the products (e.g., no additives)
		Product safety: the product is safe technically
		Benefits: presents a message about an advantage of using the product (e.g., “no smell”, “not gross”, “good smell”, and “customizable device”)
		Durability: Information about usage life of e-cigarette product
		History: illustrate the brand/product history as endorsement for its quality claim
		Country of origin: made in the USA, made locally
		Comparison with other e-cigarettes: compares product to other electronic cigarette product brands directly or indirectly (e.g., “best”, “a pinch better”, “there’s no other e-cigarette/brand like it”, and “better than other cigarette brands”)
**Price**	**Discount or price advantage**	Information about available market price of e-cigarette. Price conveys that using the product will save the consumer money or provide a better monetary value than using tobacco/nicotine products, or offers the user coupons/discounts (e.g., “less expensive”, “getting more for your money”, “25% off”, “cost savings”, “Free Offers”, and “free samples of product”)
**Youth**	**Youth-resonant information**	Normalizing e-cigarettes as youth-related products by placing them near the popular youth items or making them visible in youth places such as sports venues, places with video games, school, concerts, music events or at the movies, with the implication that e-cigarettes are suitable for youth
**Warnings/Disclaimers**	**Health disclaimers/warnings**	Health disclaimer/warning features the potential risk/harm of the product with/without instruction for first-aid treatment (e.g., contains nicotine; product should not be used to quit; pregnant/breastfeeding women should not use it; nicotine is harmful; not a treatment/cessation device; specific health conditions; ingestion/contact with skin; animal warning; state-specific warning; nicotine overdose symptoms; recommends user quit smoking; not FDA approved or regulated; may contain traces of nuts; harmful to aquatic organisms and environment; if you are on medicine, consult your GP; nicotine is highly addictive; E-cigarette is a smoking product; e-cigarettes are not approved as smoking cessation devices (e.g., not smoking cession device); etc.)
	**Age restriction**	The product will not be sold to people under the age of 18 or to minors (underage warning/disclaimer or pop-up window, etc.)
**Purchase information**	**Information for easy purchase**	Information about distribution channel of e-cigarettes, indicating the ease of accessibility 1. Website directs consumer to a website, Facebook page, social media site, or nonlocal number (i.e.,1–800) 2. Location Directs consumer to a local phone number or location (i.e., physical address)
**Others**	**Miscellaneous messages**	Information such as e-cigarette targeting women markets and e-cigarette related news and law, etc.

## Appendix C

**Table A3:** Domains of risk of bias assessment.

Domains	Description	Judgement
methods for sample selection	Appropriateness of the sampling method	Is the sample selection method free of selection bias?
methods for data collection	Appropriateness of the method used to collect data	Is the method used to collect data appropriate to avoid putting the study at a high risk of bias?
methods for data analysis	Appropriateness of the method used to analyze data	Is the method used to analyze data appropriate to avoid putting the study at a high risk of bias?
selective reporting	Possibility of selective outcome reporting	Are reports of the study free of suggestion of selective outcome reporting?
conflict of interest	declarations of conflict of interest or identification of funding sources	Is the study apparently free of conflict of interest that could put it at a high risk of bias?
other sources of bias	Any important concerns about bias not addressed in the other domains in the tool	Is the study apparently free of other problems that could put it at a high risk of bias?

## References

[1] Business Wire Global E-Cigarette Market (2022 to 2027)—Industry Trends, Share, Size, Growth, Opportunity and Forecasts. https://www.businesswire.com/news/home/20220401005272/en/Global-E-Cigarette-Market-2022-to-2027---Industry-Trends-Share-Size-Growth-Opportunity-and-Forecasts--ResearchAndMarkets.com#:~:text=The%20global%20e%2Dcigarette%20market,4.5%25%20during%202022%2D2027.

[2] Cantrell J., Emelle B., Ganz O., Hair E.C., Vallone D. (2016). Rapid increase in e-cigarette advertising spending as Altria’s MarkTen enters the marketplace. Tob. Control.

[3] Kim A.E., Arnold K.Y., Makarenko O. (2014). E-cigarette advertising expenditures in the U.S., 2011–2012. Am. J. Prev. Med..

[4] Kornfield R., Huang J., Vera L., Emery S.L. (2015). Rapidly increasing promotional expenditures for e-cigarettes. Tob. Control.

[5] Kotler P., Lane K.K. (2009). Marketing Management.

[6] Collins L., Glasser A.M., Abudayyeh H., Pearson J.L., Villanti A.C. (2019). E-Cigarette Marketing and Communication: How E-Cigarette Companies Market E-Cigarettes and the Public Engages with E-cigarette Information. Nicotine Tob. Res. Off. J. Soc. Res. Nicotine Tob..

[7] Sebastian M. E-Cig Marketing Budgets Growing by More than 100% Year over Year. https://adage.com/article/media/e-cig-companies-spent-60-million-ads-year/292641.

[8] Oster E. Juul Halts Most U.S. Advertising After Spending $104 Million in First Half of 2019. https://www.adweek.com/brand-marketing/juul-halts-mosts-u-s-advertising-after-spending-104-million-in-first-half-of-2019/.

[9] Camenga D., Gutierrez K.M., Kong G., Cavallo D., Simon P., Krishnan-Sarin S. (2018). E-cigarette advertising exposure in e-cigarette naïve adolescents and subsequent e-cigarette use: A longitudinal cohort study. Addict. Behav..

[10] Pokhrel P., Fagan P., Kehl L., Herzog T.A. (2015). Receptivity to E-cigarette Marketing, Harm Perceptions, and E-cigarette Use. Am. J. Health Behav..

[11] Giovenco D.P., Casseus M., Duncan D.T., Coups E.J., Lewis M.J., Delnevo C.D. (2016). Association Between Electronic Cigarette Marketing Near Schools and E-cigarette Use Among Youth. J. Adolesc. Health.

[12] Pepper J.K., Emery S.L., Ribisl K.M., Southwell B.G., Brewer N.T. (2014). Effects of advertisements on smokers’ interest in trying e-cigarettes: The roles of product comparison and visual cues. Tob. Control.

[13] Mantey D.S., Cooper M.R., Clendennen S.L., Pasch K.E., Perry C.L. (2016). E-Cigarette Marketing Exposure Is Associated with E-Cigarette Use Among US Youth. J. Adolesc. Health.

[14] Vassey J., Valente T., Barker J., Stanton C., Li D., Laestadius L., Cruz T.B., Unger J.B. (2022). E-cigarette brands and social media influencers on Instagram: A social network analysis. Tob. Control.

[15] Vogel E.A., Ramo D.E., Rubinstein M.L., Delucchi K.L., Darrow S.M., Costello C., Prochaska J.J. (2020). Effects of Social Media on Adolescents’ Willingness and Intention to Use E-Cigarettes: An Experimental Investigation. Nicotine Tob. Res..

[16] Cheung M.L., Leung W.K.S., Aw E.C.-X., Koay K.Y. (2022). “I follow what you post!”: The role of social media influencers’ content characteristics in consumers’ online brand-related activities (COBRAs). J. Retail. Consum. Serv..

[17] Vrontis D., Makrides A., Christofi M., Thrassou A. (2021). Social media influencer marketing: A systematic review, integrative framework and future research agenda. Int. J. Consum. Stud..

[18] D’Angelo H., Rose S.W., Golden S.D., Queen T., Ribisl K.M. (2020). E-cigarette availability, price promotions and marketing at the point-of sale in the contiguous United States (2014–2015): National estimates and multilevel correlates. Prev. Med. Rep..

[19] Kong G., Kuguru K.E., Bhatti H., Sen I., Morean M.E. (2021). Marketing Content on E-Cigarette Brand-Sponsored Facebook Profile Pages. Subst. Use Misuse.

[20] Ali F.R.M., Marynak K.L., Kim Y., Binns S., Emery S.L., Gomez Y., King B.A. (2020). E-cigarette advertising expenditures in the USA, 2014–2018. Tob. Control.

[21] Dai H., Hao J. (2017). Direct Marketing Promotion and Electronic Cigarette Use Among US Adults, National Adult Tobacco Survey, 2013–2014. Prev. Chronic. Dis..

[22] Krugman D.M. (2016). Understanding the Impact That Marketing, Advertising, and Promotion Have on Adolescent E-cigarette Behavior. J. Adolesc. Health.

[23] U.S. Food & Drug Administration FDA Takes Significant Steps to Protect Americans from Dangers of Tobacco through New Regulation. https://www.fda.gov/news-events/press-announcements/fda-takes-significant-steps-protect-americans-dangers-tobacco-through-new-regulation.

[24] U.S. Food & Drug Administration FDA Permits Marketing of E-Cigarette Products, Marking First Authorization of Its Kind by the Agency. https://www.fda.gov/news-events/press-announcements/fda-permits-marketing-e-cigarette-products-marking-first-authorization-its-kind-agency.

[25] Kotler P., Keller K.L. (2012). Marketing Management.

[26] Hutton J.G. (1999). The definition, dimensions, and domain of public relations. Public Relat. Rev..

[27] Ukaj F. (2016). Public Relations as Part of Integrated Communication of an Enterprise-Consumer Oriented On. J. Mark. Manag..

[28] Kotler P., Keller K.L., Brady M., Goodman M., Hansen T. (2009). Marketing Management.

[29] Katrak P., Bialocerkowski A.E., Massy-Westropp N., Kumar V.S.S., Grimmer K.A. (2004). A systematic review of the content of critical appraisal tools. BMC Med. Res. Methodol..

[30] Moja L.P., Telaro E., D’Amico R., Moschetti I., Coe L., Liberati A. (2005). Assessment of methodological quality of primary studies by systematic reviews: Results of the metaquality cross sectional study. BMJ Clin. Res..

[31] Nagpal J., Kumar A., Kakar S., Bhartia A. (2010). The development of Quality of Life Instrument for Indian Diabetes patients (QOLID): A validation and reliability study in middle and higher income groups. J. Assoc. Physicians India.

[32] Higgins J.P., Altman D.G. (2008). Assessing Risk of Bias in Included Studies. Cochrane Handbook for Systematic Reviews of Interventions.

[33] Sanderson S., Tatt I.D., Higgins J.P. (2007). Tools for assessing quality and susceptibility to bias in observational studies in epidemiology: A systematic review and annotated bibliography. Int. J. Epidemiol..

[34] Hartmann-Boyce J., McRobbie H., Lindson N., Bullen C., Begh R., Theodoulou A., Notley C., Rigotti N.A., Turner T., Butler A.R. (2020). Electronic cigarettes for smoking cessation. Cochrane Database Syst. Rev..

[35] McNeill A.B.L., Calder R., Simonavicius E., Robson D. (2021). Vaping in England: An Evidence Update Including Vaping for Smoking Cessation.

[36] Siu A.L. (2015). Behavioral and Pharmacotherapy Interventions for Tobacco Smoking Cessation in Adults, Including Pregnant Women: U.S. Preventive Services Task Force Recommendation Statement. Ann. Intern. Med..

[37] U.S. Food & Drug Administration Fact or Fiction: What to Know About Smoking Cessation and Medications..

[38] Royal College of Physicians (2019). RCP Advice on Vaping Following Reported Cases of Deaths and Lung Disease in the US.

[39] National Academies of Sciences, Engineering, and Medicine (2018). Public Health Consequences of E-Cigarettes.

[40] American College of Physicians (ACP) ACP: Tighter Regulations, More Research Needed on E-Cigarettes and Their Consequences. https://www.acponline.org/advocacy/acp-advocate/archive/november-15-2019/acp-tighter-regulations-more-research-needed-on-e-cigarettes-and-their-consequences.

[41] Yates K., Friedman K., Slater M.D., Berman M., Paskett E.D., Ferketich A.K. (2015). A Content Analysis of Electronic Cigarette Portrayal in Newspapers. Tob. Regul. Sci..

[42] Lyu J.C., Wang D., Huang P., Ling P. (2021). News Media Coverage of E-Cigarettes: An Analysis of Themes in Chinese Newspapers. Int. J. Commun..

[43] Mackey T.K., Miner A., Cuomo R.E. (2015). Exploring the e-cigarette e-commerce marketplace: Identifying Internet e-cigarette marketing characteristics and regulatory gaps. Drug Alcohol Depend..

[44] Huang J., Kornfield R., Szczypka G., Emery S.L. (2014). A cross-sectional examination of marketing of electronic cigarettes on Twitter. Tob. Control.

[45] Paek H.J., Kim S., Hove T., Huh J.Y. (2014). Reduced harm or another gateway to smoking? Source, message, and information characteristics of E-cigarette videos on YouTube. J. Health Commun..

[46] McCausland K., Maycock B., Leaver T., Jancey J. (2019). The Messages Presented in Electronic Cigarette-Related Social Media Promotions and Discussion: Scoping Review. J. Med. Internet. Res..

[47] Belch G.E., Belch M.A. (2004). Advertising and Promotion: An Integrated Marketing Communications Perspective.

[48] Lewis M.J., Ling P.M. (2016). “Gone are the days of mass-media marketing plans and short term customer relationships”: Tobacco industry direct mail and database marketing strategies. Tob. Control.

[49] Kliatchko J. (2005). Towards a new definition of Integrated Marketing Communications (IMC). Int. J. Advert..

[50] Delnevo C.D., Jeong M., Teotia A., Bover Manderski M.M., Singh B., Hrywna M., Wackowski O.A., Steinberg M.B. (2022). Communication Between US Physicians and Patients Regarding Electronic Cigarette Use. JAMA Netw. Open.

[51] U.S. Preventive Services Task Force Tobacco Smoking Cessation in Adults, Including Pregnant Persons: Interventions. https://www.uspreventiveservicestaskforce.org/uspstf/recommendation/tobacco-use-in-adults-and-pregnant-women-counseling-and-interventions.

[52] Shapiro H. (2018). No Fire, No Smoke: The Global State of Tobacco Harm Reduction. https://gsthr.org/resources/thr-reports/no-fire-no-smoke-global-state-tobacco-harm-reduction-2018/.

[53] Anderson S.J., Dewhirst T., Ling P.M. (2006). Every document and picture tells a story: Using internal corporate document reviews, semiotics, and content analysis to assess tobacco advertising. Tob. Control.

